# Axial Compression Behaviours of Pultruded GFRP–Wood Composite Columns

**DOI:** 10.3390/s19040755

**Published:** 2019-02-13

**Authors:** Yujun Qi, Lei Xie, Yu Bai, Weiqing Liu, Hai Fang

**Affiliations:** 1 College of Civil Engineering, Nanjing Tech University, Nanjing 211816, China; wqliu@njtech.edu.cn (W.L.); fanghainjut@163.com (H.F.); 2 Department of Civil Engineering, Monash University, Clayton 3800, Australia; lei.xie@monash.edu (L.X.); yu.bai@monash.edu (Y.B.)

**Keywords:** fiber reinforced polymer (FRP), pultrusion process, composite column, slenderness ratio, buckling

## Abstract

An innovative pultruded fiber reinforced polymer (FRP)–wood composite (PFWC) column with a lightweight southern pine wood core confined by outer FRP sheets was manufactured using an improved pultrusion process. Axial compression tests with both ends pinned as boundary conditions were employed to investigate the mechanical performance of such PFWC columns under concentric load. Through experimental investigations, the effects of the slenderness ratio on the failure modes and the axial load bearing capacities of the PFWC columns were evaluated. The failure modes showed that the specimens with a slenderness ratio less than 43.2 failed through compressive failure at junctions on FRP sheets, while those with slenderness ratios larger than 57.6 showed global buckling. Strain responses on specimens with different slenderness ratios are consistent with the observed failure modes. Finite element analysis was carried out to validate the experimental results, and satisfactory agreement was found between the failure modes and load–displacement curves. An empirical equation was developed with a new factor taking 0.65 into account to predict the load bearing capacities of the PFWC columns, and good agreement was found.

## 1. Introduction

Currently, pultruded fiber-reinforced polymer (FRP) profiles are increasingly used in structural engineering as main structural components, such as girders, decks, and columns [1,2,3,4,5,6], because of their advantages of high strength-to-weight ratio, high corrosion prevention, convenient use and decreasing costs. Common pultruded FRP profiles with thin-wall sections, such as I-sections and box-sections, may experience local/global buckling under bending or compression [7,8,9,10] and delamination failure in the web-flange junction region during post-buckling phase [11,12,13,14,15]. To overcome the stability issue with FRP thin-wall sections, FRP sandwich structures with lightweight core materials may perform better than a pultruded FRP profile with thin-wall sections due to the enhanced flexural stiffness [16,17].

FRP sandwich structures are composed of FRP face sheets and lightweight core materials, such as foam, honeycomb, and lightweight woods. The axial compressive behaviours of FRP sandwich structures have been investigated in a large number of experimental and theoretical studies [18,19,20]. For example, Fleck and Sridhar [18] studied the failure modes of sandwich columns with glass fiber-reinforced polymer (GFRP) sheets and PVC foam cores, Veedu and Carlsson [19] established a finite element model to analyze the buckling of sandwich columns containing a face/core de-bonding, and Mitra and Raja [20] improved an innovative methodology of inserting pre-manufactured shear keys in foam core grooves to increase the delamination resistance capacity of a sandwich composite column. The sandwich columns in their research are common sandwich panels with two-sided FRP face sheets and a core located between two FRP face sheets under in-plane compression. Compared with this sandwich column, the sandwich columns with inner cores and FRP sheets wrapped on all of the outer faces of the column have different mechanical performances. Numerous experimental and theoretical studies on the load-bearing capacities of pultruded FRP profiles without core materials have been conducted in the past two decades [21,22,23,24,25,26,27,28]. Barbero and Tomblin [21] proposed a design equation for pultruded GFRP profiles with wide-flange I-sections. An empirical constant named the mode-interaction constant is used to account for the interaction between global buckling and local buckling and is suggested to be 0.84. Subsequently, the mode-interaction constant was set to 0.65 in their research [22]. Lane and Mottram [23] indicated that the 0.65 value may be conservative, and a value of 0.80 was recommended by Bank [24]. Hashem [25] conducted experimental and analytical investigations on short GFRP composite compression members with “universal” and “box” cross sections. The failure modes included local flange buckling and compressive strength failures. In addition, the unstiffened flanges of the columns were analyzed with various boundary conditions using classical orthotropic plate theory and the finite element (FE) method. Turvey and Zhang [26] proposed a two-dimensional FE model for predicting the buckling, post-buckling, and initial failure loads of pultruded GFRP wide-flange (WF) columns. In 2006, Puente et al. [27] proposed a new design equation for pultruded GFRP profiles, following the approach adopted by Eurocode3 for steel columns. In this equation, the coefficient γ accounts for the quality of the material supplied by a manufacturer to reduce the value of the critical load, and γ = 1.2 was suggested. Bai and Keller [28] proposed a formulation for predicting the ultimate load of pultruded FRP columns based on shear failure and second-order deformation, and the effects of initial imperfections, slenderness, the shear-to-compressive strength ratio, the shear coefficient, and the type of shear failure criterion on the ultimate load and failure mode (shear or compressive failure) were studied.

**Figure 1:**
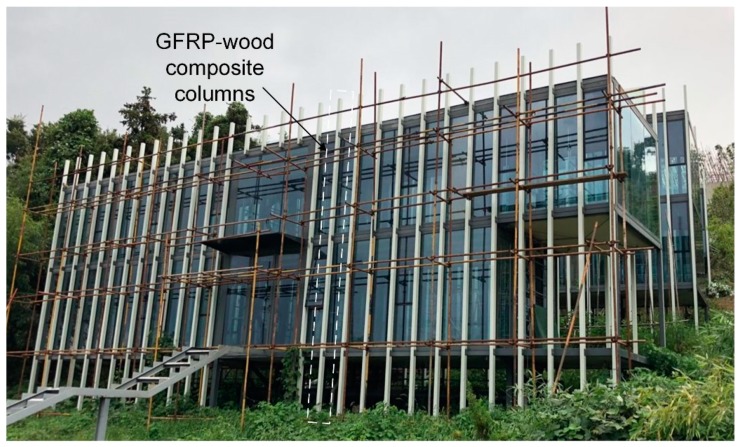
The Nanjing Foshou Lake Hotel building with GFRP–wood composite column applications.

The manufacturing process of FRP sandwich structures is often assisted by a hand lay-up process or resin infusion process (RIP) [16], which can result in lower efficiency and quality and higher unit production costs than compared with the pultrusion process. Therefore, the pultrusion process of FRP sandwich structures has been investigated, and results of the FRP sandwich structures made by the pultrusion process have been reported in the literature. McGrath [29] applied for a patent for continuously manufacturing a composite sandwich structure by pultrusion, and Fanucci [30] applied for a patent for a pultrusion method that produces a composite structural member with embedded rigid elements. Patrick [31] studied the shear and flexural performances of a proposed 3D sandwich panel that was fabricated using a pultrusion process. Dawood [32] subsequently studied the static and fatigue bending behaviour of pultruded GFRP sandwich panels with through-thickness fiber insertions. Another relevant work is from West Virginia University, Morgantown, WV, USA, which studied FRP sandwich panels with a wood core produced using the pultrusion process (www.cemr.wvu.edu). However, there is no detailed investigation reported on such pultruded sandwich core panels. Recently, the Nanjing Foshou Lake Hotel building, which is a whole-FRP composite building [33], was constructed in Nanjing, China. In this three-storey building, FRP–wood composite columns are employed to formulate condensed structures (see Figure 1) to resist vertical loads; such composite columns (with section dimensions of 120mm × 90 mm) are used throughout the three storeys, and the longest one is 11.1 m. This type of FRP–wood composite (PFWC) profile is fabricated by the pultrusion process and has an inner wood core and outer FRP sheets. In the present study, to thoroughly understand the mechanical behaviors of the proposed FRP–wood composite profile for column application, axial compression tests were employed, and the failure modes and critical load capacities were obtained. Finite element analysis was also employed to validate the experimental results, including failure modes and load–displacement curves. Finally, the load bearing capacities of such PFWC profiles were examined with different design methods, and an improved empirical equation was developed.

## 2. The Manufacturing Process

**Figure 2:**
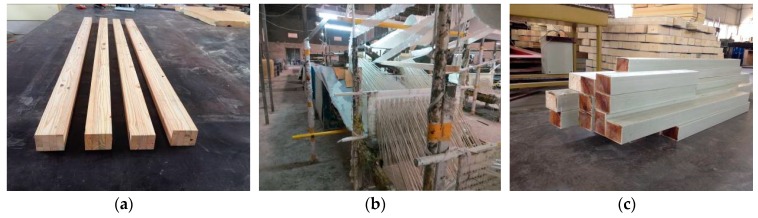
Manufacturing of PFWC column specimens: (**a**) southern pine wood as core material, (**b**) an improved pultrusion process, and (**c**) completed column specimens.

The PFWC column was manufactured by an improved pultrusion process in Nangjing Spare Composites CO. LTD, Nanjing, China. E-glass fiber and unsaturated polyester resin (UPR) were used for the glass FRP (GFRP) skins, and chopped strand mats composed of glass fibers were used on the inside and outside surfaces of the GFRP skins. As shown in Figure 2, the improved pultrusion process for the PFWC profile can be divided into the following three steps: (i) The southern pine wood was cut into the designed core shape for the wood core with a rectangular section and the required length after the drying process. Then, the silane coupling agent was brushed onto the faces of the wood core (see Figure 2a). (ii) The wood core, the fibers saturated with UPR, and the mats were continuously fed into a metal mould together, and the PFWC profile was pulled out from the other end of the metal mould after the UPR cured (see Figure 2b). (iii) After cutting the continuous PFWC profiles, the manufacture of the PFWC columns was completed (see Figure 2c). The volume fraction of the glass fiber in this FRP/wood composite columns is 65–68%.

## 3. The Experimental Program

### 3.1. Materials

The southern pine wood was employed as the core material in this study. Six cubic wood coupons were constructed and tested in compression according to ASTM C365/C365M-11 (Laboratory of Composite Structures, Nanjing Tech University, Nanjing, China) [34]. Compressive tests were performed to determine the properties of the pultruded GFRP skin, including the Young’s modulus and ultimate compression strength in the pultrusion direction. The coupons were taken from the GFRP skin of the PFWC profile and tested according to ASTM D 695-10 [35] for tensile properties. The testing results of wood and GFRP coupons are presented in Table 1. The measured compressive modulus and strength of the southern pine wood were 7.4 GPa and 51.8 MPa, and those of the GFRP skin in the pultrusion direction were 28.0 GPa and 163.2 MPa, respectively.

### 3.2. Specimens

**Figure 3:**
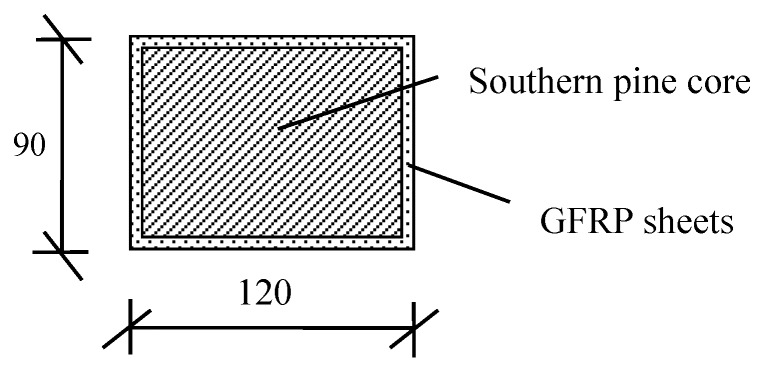
Section of a pultruded GFRP–wood composite column.

The GFRP–wood composite columns tested in this study were cut from the PFWC profile with a rectangular cross section of 120 mm × 90 mm manufactured by the above improved pultrusion process. The cross section of the southern pine wood core was 110 mm × 80 mm, and the external GFRP sheets were 5 mm thick as shown in Figure 3. The fiber direction of the GFRP sheets was consistent with the pultrusion direction, and the wood grain of the southern pine wood core was consistent with the fiber direction, i.e., perpendicular to the cross section of the composite column profile.

A total of 12 GFRP–wood composite column specimens with six different lengths were tested under axial compression loading, as listed in Table 2. Two specimens were tested for each length. All of the specimens had the same section shown in Figure 3. The details of the specimens are given in Table 2.

The slenderness ratio λ of the GFRP–wood composite column specimens was calculated using the following equation:

(1) $$\lambda =\frac{L_{\mathrm{cr}}}{r}$$

where *L*_cr_ is the effective length of the specimens, and r is the radius of gyration for the section of the specimens, which can be calculated by

(2) $$r=\sqrt{\frac{I_{\mathrm{total}}}{A_{\mathrm{total}}}}=\sqrt{\frac{I_{f}+\alpha_{E}I_{w}}{A_{f}+\alpha_{E}A_{w}}}$$

where *I*_f_ and *I*_w_ are the cross-sectional moments of inertia for the GFRP sheet area and the southern pine core area, *A*_f_ and *A*_w_ are the areas for the GFRP sheet section and the southern pine core section, respectively, and *α*_E_ is the ratio of the Young’s moduli between the southern pine and the GFRP sheet.

### 3.3. Test Set-Up and Instrumentation

**Figure 4:**
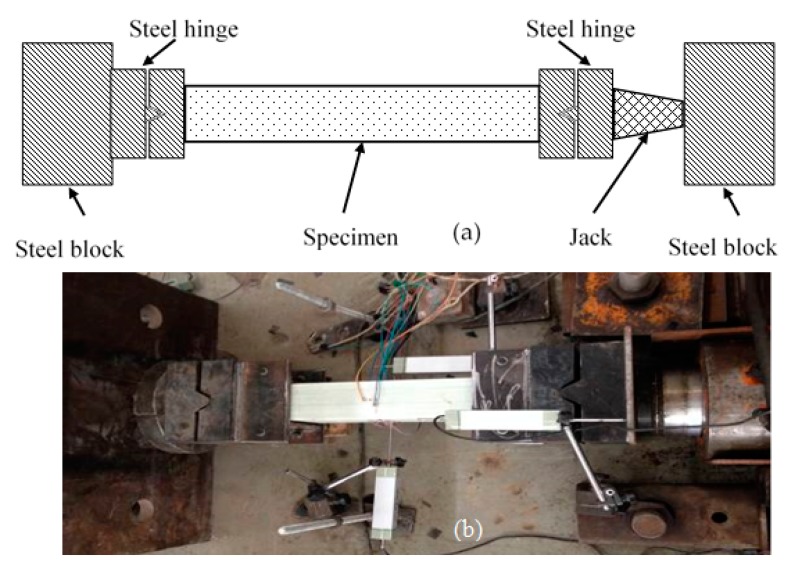
Test setup: (**a**) schematic diagram and (**b**) photograph.

The compression tests were performed on a special setup, as shown in Figure 4. The support system consisted of two strong steel blocks bolted to the floor. Loading was applied by a hydraulic actuator with an axial capacity of 1000 kN. The specimen was placed inside the two steel blocks. Both ends of the column specimen were connected to special steel hinges, respectively, and one of the steel hinges was connected to the hydraulic actuator. The steel hinge was composed of two pieces of thick steel panel: one had a hump and the other had the corresponding groove, as shown in Figure 4a,b.

**Figure 5:**
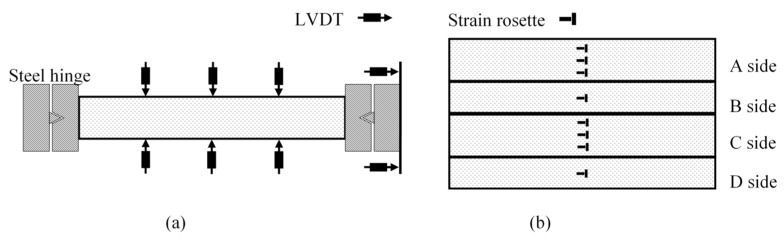
Instrumentation: (**a**) linear variable displacement transducers (LVDTs) and (**b**) strain gauges.

Eight linear variable displacement transducers (LVDTs) with a stroke of 100 mm were used to measure the displacements of the column under compression. Two of the LVDTs were set along the column to measure the axial shortening of specimen, and three LVDTs were installed on each side of the specimen to measure the horizontal deflections at each quarter of the specimen, as shown in Figure 5a.

For each specimen, the strains in the GFRP skins were measured using electrical resistance linear strain gauges. The strain gauges were attached to the GFRP skins at the mid-height of the specimens. On the two wide faces of the specimen, six bi-directional strain rosettes with a gauge length of 20 mm were evenly distributed, and two identical strain rosettes were set on the two narrow faces of the specimen to measure the axial and lateral strains, respectively. The locations of the strain rosettes are shown in Figure 5b. The measured strains were then used to identify the failure mode.

The axial compressive loading was applied in displacement control at a loading rate of 2 mm/min for all of the specimens. The tests were stopped when the load of the specimen reduced by at least 20% of the maximum load of the specimen.

## 4. Results and Discussion

### 4.1. Axial Compressive Behaviour and Failure Mode

#### 4.1.1. Compressive Failure

**Figure 6:**
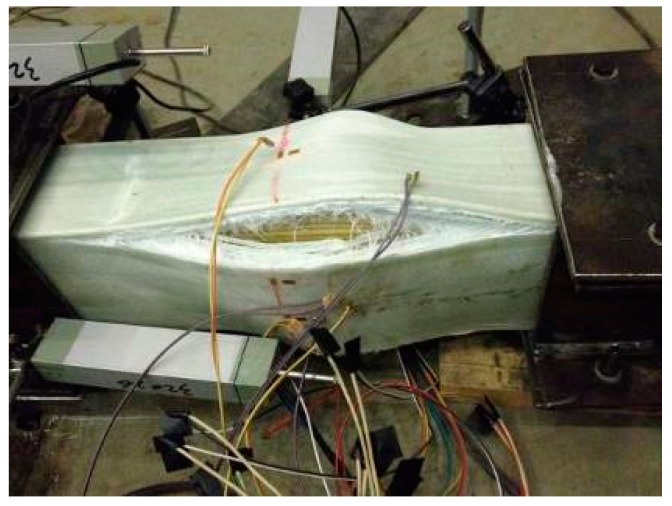
Failure mode of PC-500-1.

**Figure 7:**
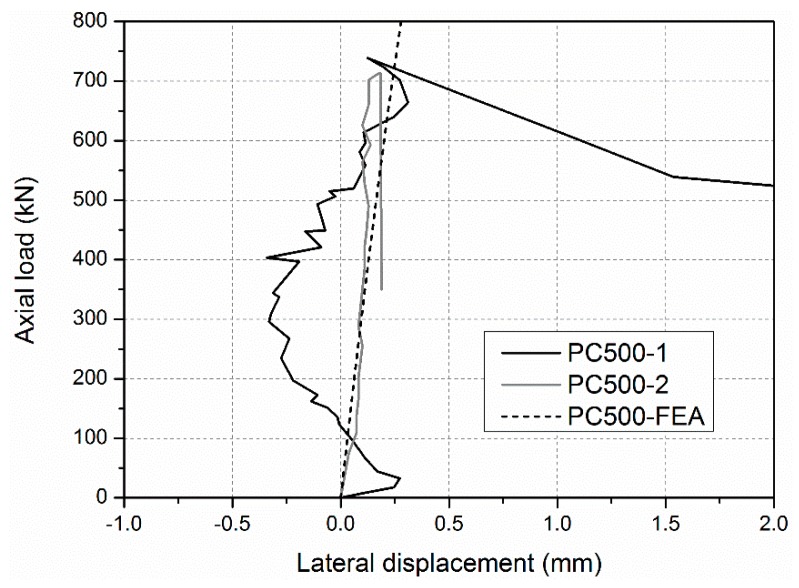
Lateral deformations of PC-500 specimens.

**Figure 8:**
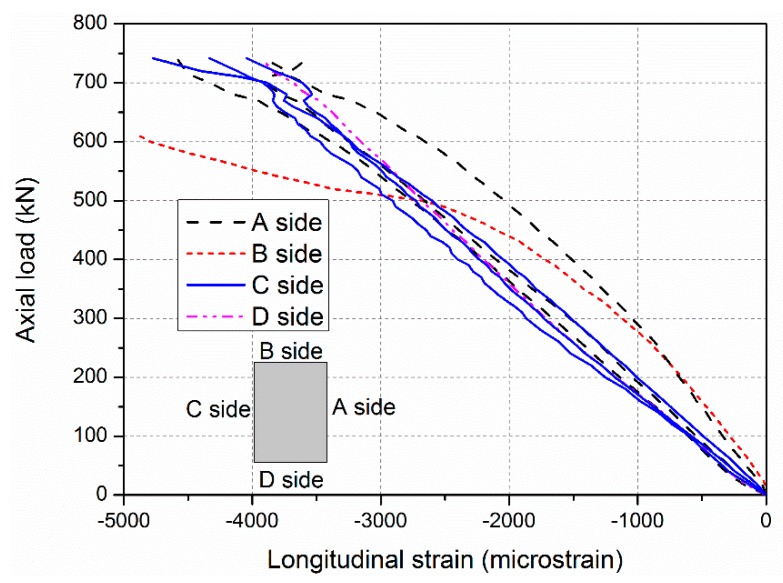
Longitudinal strains of the GFRP sheets of the specimen PC-500-1.

During loading procedure, no visible phenomena were observed in the specimens PC-500-1 and PC-500-2, firstly. Then slight cracking sounds were heard intermittently before reaching the ultimate axial load capacity. The applied load decreased significantly when a big bang suddenly occurred. The GFRP skins bulged out, and the junction between the adjacent GFRP sheets cracked, as shown in Figure 6. Before the failure occurred, the lateral deformations of specimens PC-500-1 and PC-500-2 were small (less than 0.36 mm), recorded by LVDTs in the tests, as shown in Figure 7. Meanwhile, all the longitudinal strains of GFRP sheets were increased linearly with the increasing compressive load, as shown in Figure 8, and the compressive strains reached more than −0.0047, 0.81 times higher than the ultimate compressive strain, when the junction between the adjacent side GFRP sheets cracked and the GFRP sheets bulged out. This evidence indicates a typical compressive failure for a short sandwich column.

#### 4.1.2. Global Buckling

**Figure 9:**
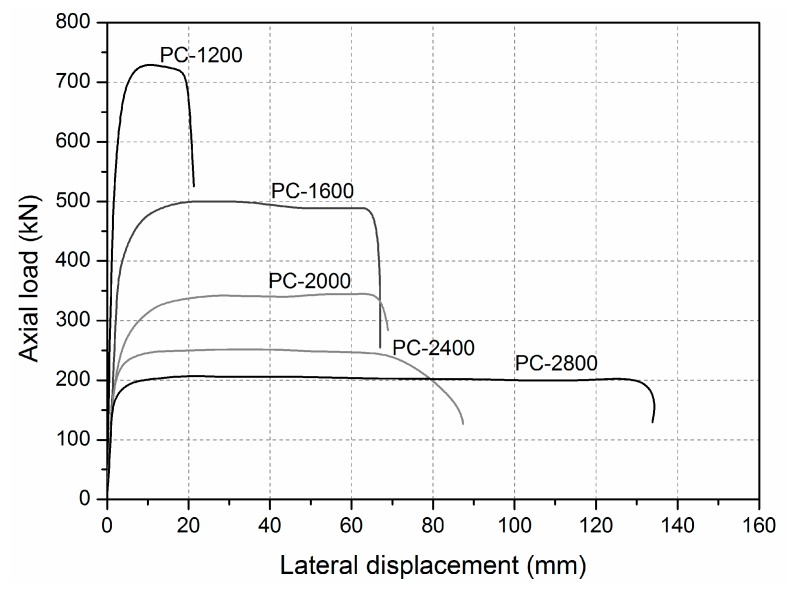
Lateral deformation of specimens ranging from 1200 to 2800 mm in length.

**Figure 10:**
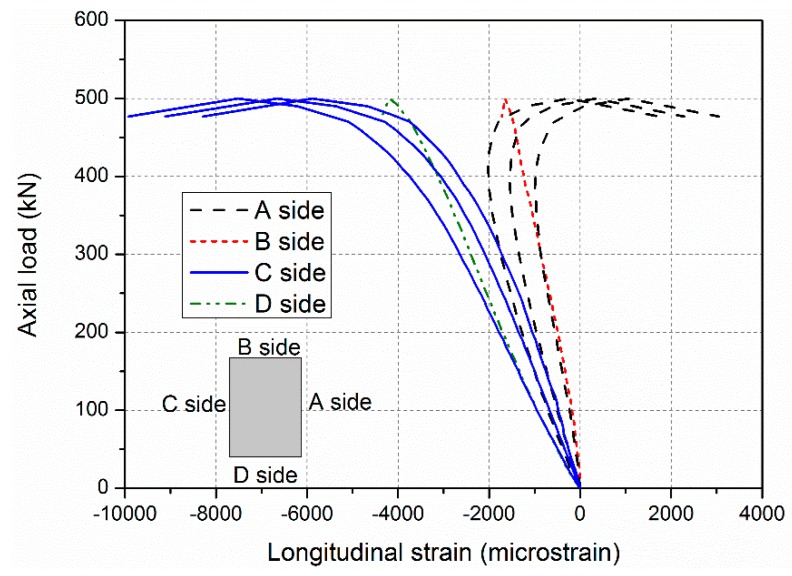
Longitudinal strains of the GFRP sheets of the specimen PC-1600-1.

Global buckling was found in specimens ranging from 1600 to 2800 mm in length. Similar load–lateral deformation behaviour was observed in all specimens shown in Figure 9. During the loading procedure, the lateral deformation of specimen was first small and then increased rapidly with a small increasing compressive load. Meanwhile, similar load–longitudinal strain curves were found in specimens ranging from 1600 to 2800 mm in length. For example, in the specimen PC-1600, as shown in Figure 10, all the longitudinal strains of all sides of the GFRP sheets increased linearly before the load reached 500 kN. The absolute values of the longitudinal strains of the GFRP sheets on the A side then reduced rapidly. Those on the C side continued to increase, but the compressive load could not increase. This indicated that an obvious transverse deformation occurred in the specimen, which led to compression on the A side and to tensile on the C side. This specimen was thusfailed in global buckling. It should be noted that no visible failure in the GFRP sheets was observed when the global buckling failure of this specimen occurred.

#### 4.1.3. Compressive Failure/Global Buckling Interaction

**Figure 11:**
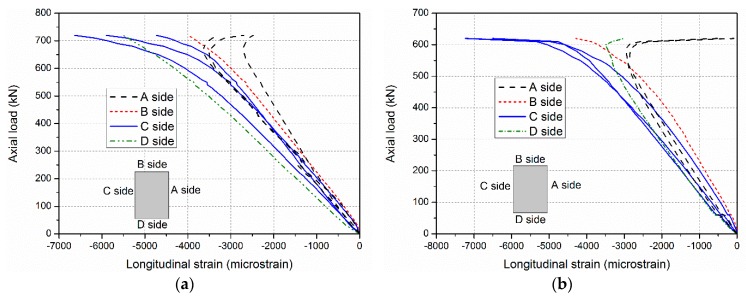
Longitudinal strains of the GFRP sheets of the specimens (**a**) PC-1200-1 and (**b**) PC-1200-2.

**Figure 12:**
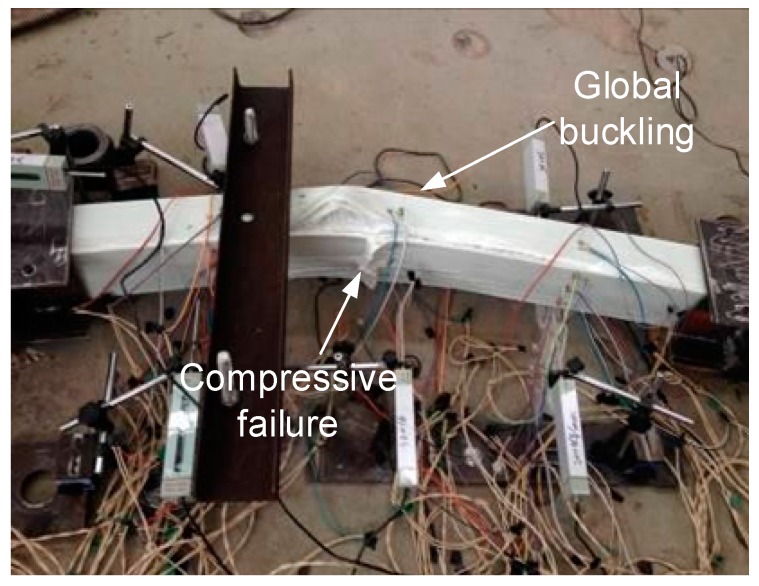
Combined compressive failure/global buckling mode of the specimen PC-1200-1.

For intermediate length specimens PC-1200-1 and PC-1200-2, combined modes of compressive failure and global buckling were found. In the load–lateral displacement curves shown in Figure 9, lateral displacement is evident (around 20 mm). A flat region indicates the global buckling mode. The ultimate load capacities are 730.2 and 719.8 kN, which is close to the ultimate section capacities (717.0 and 741.4 kN based on the test results of the PC-500 series). Load–longitudinal strain relationships are presented in Figure 11. The A and C sides gradually bifurcated after the axial loads passed 650 kN, indicating global buckling, which led to the overall lateral deformation. The failure modes also prove the compressive failure/global buckling interaction mode. Figure 12 shows the compressive failure, while the obvious lateral deformations (larger than 20 mm) indicate that global buckling mode occurred in the meantime.

#### 4.1.4. Post-Buckling Behaviours

**Figure 13:**
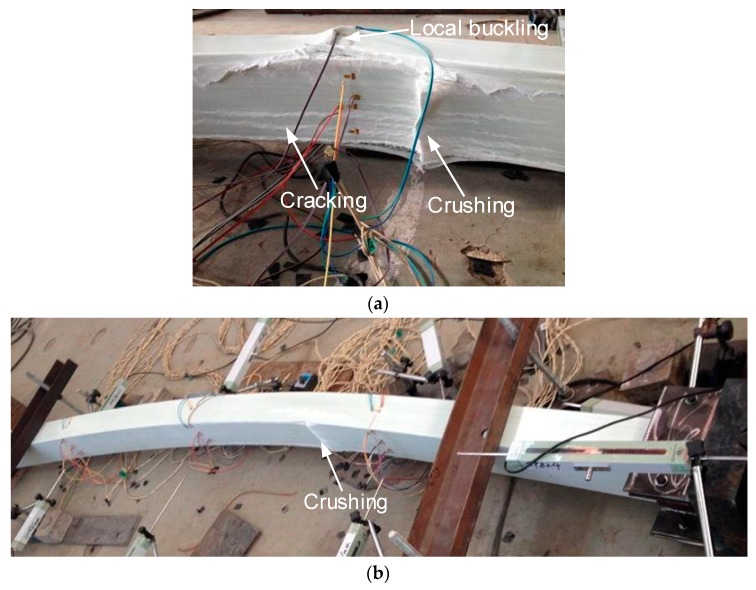
Post-buckling failure of specimens (**a**) PC-1600-2 and (**b**) PC-2800-2.

The loading processes continued after the global buckling of specimens occurred, to investigate the post-buckling behaviours of specimens under concentric loading. During the subsequent loading process, the lateral deformation of specimens increased dramatically, with an almost invariable load until the final failure occurred. The basic final failure modes include three actions: (1) crushing of the compressive GFRP sheet, (2) cracking of the compressive GFPR sheet in the pultrusion direction, and (3) local buckling of the side GFRP sheets with cracking of the web–flange junctions. The real final failure modes of specimens PC-1600 and PC-2000 with shorter length were a combination of the above basic final failure modes (1), (2), and (3), as shown in Figure 13a, and the following sequence: First, longitudinal cracking in the pultrusion direction occurred on the GFRP sheets, and the compressive GFRP sheet was then crushed in the middle; after that, the stress migrated to the side GFRP sheets and the wood core, leading to their debonding; after the debonding, local buckling then occurred on the side GFRP sheets. Specimens PC-2400 and PC-2800 with longer lengths were mainly a single basic failure mode (1), as shown in Figure 13b. This phenomenon indicated that the failure mechanism in intermediate length specimens is more complicated than that of slender specimens of pultruded GFRP–wood composite columns.

### 4.2. Compressive Ultimate Load P_U_

**Figure 14:**
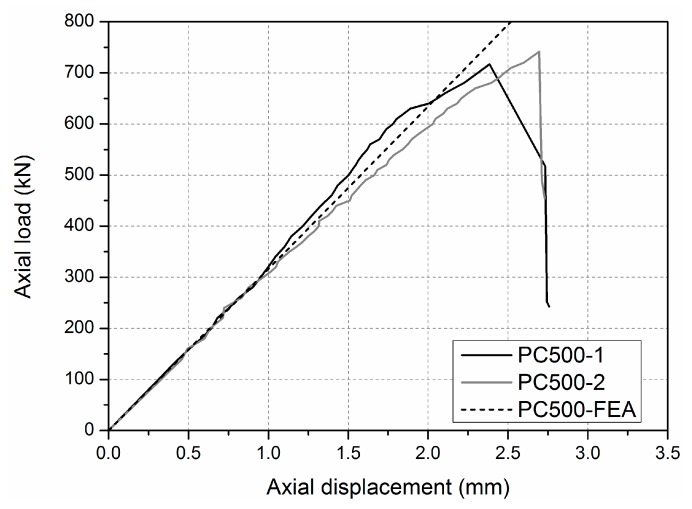
Load-axial shortening displacement curves of the PC-500 specimens.

The compressive ultimate load *P*_U_ was adopted to evaluate the bearing capacity of the specimen that experienced compressive failure mode. The load-shortening deformation curves, as shown in Figure 14, indicate that specimens PC-500-1 and PC-500-2, subjected to compressive load, remained linearly elastic until failure. Thus, the compressive ultimate load *P*_U_ could be defined by the peak compressive load, which could be found on the load–shortening deformation curve. The compressive ultimate load *P*_U_ of specimens PC-500-1 and PC-500-2 were 717.0 and 741.4 kN, with failure strains in the GFRP sheets at about 3900~4773 micro-strains recorded by gages. The calculated real stress of the GFRP sheets in the tested specimens was between 109.2 and 133.6 MPa, and the corresponding stress ratio ranged from 66.9 to 81.9%, which was defined by the ratio of the real stress of the GFRP sheets at the peak compressive load to the compressive strength of the GFRP sheets tested in Section 3.1. The phenomenon that the real stresses of the GFRP sheets at final failure status was less than its compressive strength is mainly caused by the different failure modes of column specimens comparing to the GFRP coupon specimens, as introduced in Section 4.1. This result is also found in the literature [36], but the stress ratio was lower than that of the pultruded GFRP–wood composite columns tested in this study, which ranged from 59.2 to 64.9%. This could indicate that the compressive capacity of the GFRP sheets in the GFRP–wood composite columns was more completely utilized than was that of the pure GFRP columns, because the presence of the wood core was able to prevent an inward buckling bulge, as has been reported in the literature [36].

The theoretical ultimate axial load capacity of PFWC columns can be expressed as

(3) $$P_{m}=f_{f}A_{f}+kf_{w}A_{w}$$

where *A*_f_ and *A*_w_ are the cross-sectional areas of the GFRP skins and the wood core, respectively, *f*_f_ is the axial compressive strength of the GFRP skin, and *f*_w_ is the compressive strength of the wood core. *k* is a coefficient used to consider a series of factors, including the section forms and dimensions, the height-to-width ratio of the column, and the loading rate [32]. For long columns with circular sections, *k* is assumed to be 0.67 [32]. For the short columns in this investigation, *k* is assumed to be 1.0, and the result calculated according to Equation (3) is 783.6 kN. Comparing the theoretical and experimental results indicates that the proposed theoretical model is able to conservatively estimate the actual ultimate axial load capacity of the PFWC columns under compression, with an average overestimation of 10%.

### 4.3. Critical Buckling Load P_cr_

**Figure 15:**
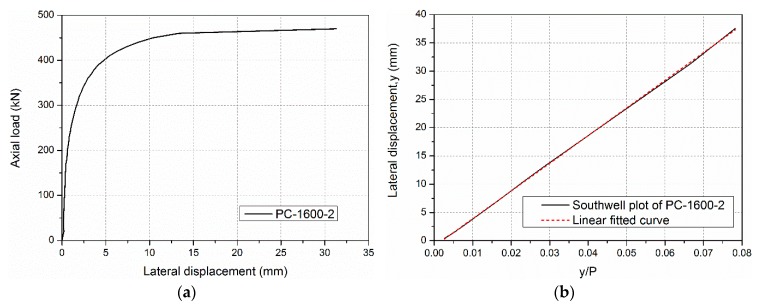
Representative results from the column test of the specimen PC-1600-2: (**a**) axial load vs. lateral displacement and (**b**) the Southwell plot.

The critical buckling load *P*_cr_ of specimens with global buckling failure in this study can be obtained from slopes in Southwell plots [22] of the mid-height lateral deflection (*y*) vs. the mid-height lateral deflection/load (*y/P*). A representative example of a column axial load vs. the lateral deflection curve and the corresponding Southwell plot is shown in Figure 15. Based on the Southwell plot, the critical buckling load *P*_cr,t_ is given as follows:

(4) $$y=P_{\mathrm{cr},t}\frac{y}{P}-a$$

where *P* is the applied axial load on the column, *y* is the mid-height lateral deflection, and *a* indicates the initial mid-height lateral deformation or the imperfection of column.

The theoretical global buckling load of ideal compressive sandwich column can be predicted by Euler critical load *P*_cr,E_ as follows:

(5) $$P_{\mathrm{cr},E}=\frac{\pi^{2}{\left(EI\right)}_{0}}{l_{0}^{2}}$$

where *l*_0_ is the effective length of the column, and (*EI*)_0_ is the bending stiffness of the sandwich column, which is the sum of the bending stiffness of the wood core and the GFRP sheet and can be calculated using Equation (6):

(6) $${\left(EI\right)}_{0}=E_{f}I_{f}+E_{w}I_{w}$$

where *E*_f_ and *E*_w_ are the elastic moduli of the GFRP sheets and the southern pine wood, and *I*_f_ and *I*_w_ are the cross-sectional moments of inertia for the GFRP sheets and the southern pine core. The critical buckling loads *P*_cr,t_ and *P*_cr,E_ calculated through Equations (4) and (5) are listed in Table 3. Table 3 also shows the ultimate loads (*P*u) of specimens in this study. The results show that the ultimate loads have a small deviation less than 10% (except 13.57% of the specimen PC-2800-2) comparing to the Euler critical load *P*_cr,E_.

### 4.4. Comparisons with FE Analysis

#### 4.4.1. Finite Element Modelling

**Figure 16:**
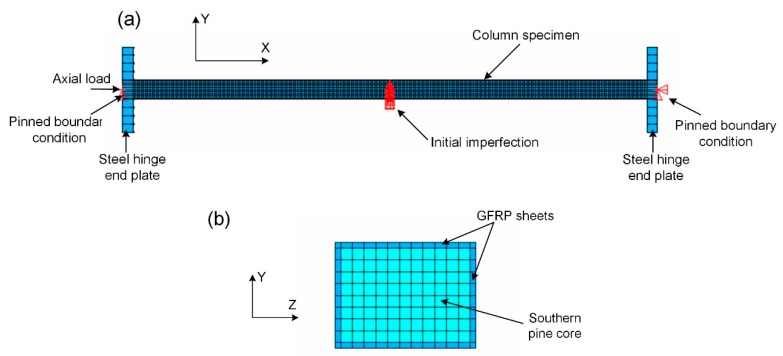
Finite element (FE) model of the GFRP–wood composite column under axial compression: (**a**) overall model; (**b**) cross section.

Numerical simulations were carried out to verify the analytical solutions and to be compared with the experimental measurements of the axial compression behaviours of the GFRP–wood composite columns. FE modelling procedures have been successfully employed in research studying the performance of FRP structures or their components. Simulations of the axial compression test of the GFRP–wood composite columns have been carried out using the ANSYS finite element program [37]. Figure 16 shows the overall numerical model used to simulate the axial compression test of the GFRP–wood composite column with a length of 2400 mm. The GFRP sheet and wood core materials of the GFRP–wood composite columns were modelled as eight-node solid element (SOLID45) with mechanical properties obtained from the coupon tests, respectively. The interface between FRP sheets and the wood core is assumed to have perfect adherence, and this is achieved by merging the coincided nodes of the GFRP sheet and wood core elements. In total, 10,080 elements of the GFRP sheets and 21,120 elements of the wood core were established. Two steel hinged plates placed on the ends of the specimen were also modelled as eight-node solid element. The boundary conditions were modelled as a pin for the end supports of two steel plates, as shown in Figure 16a. An initial imperfection equal to 1/500 of the column specimen length was introduced at mid-height, and a nonlinear analysis was further performed.

#### 4.4.2. Result Comparisons

**Figure 17:**
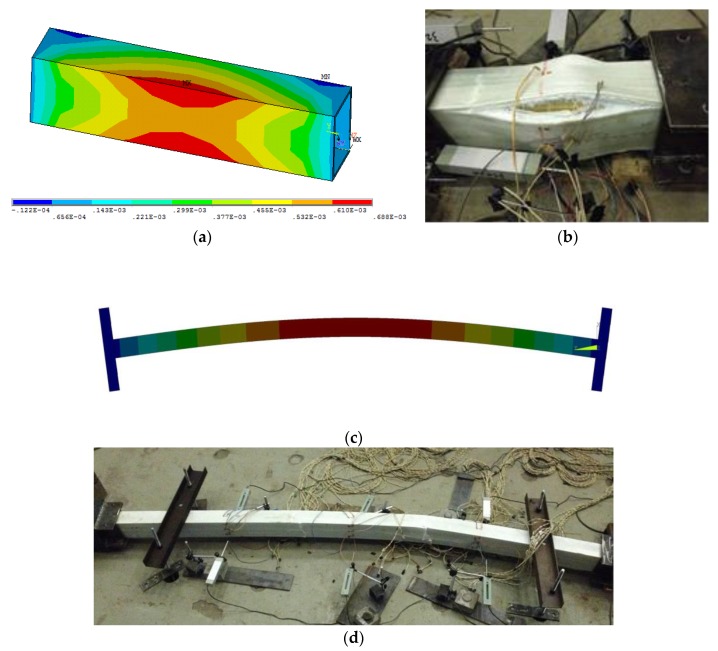
Comparison between experimental and FE modeling failure modes: (**a**) PC-500 (FE); (**b**) PC-500 (Exp); (**c**) PC-2400 (FE); (**d**) PC-2400 (Exp).

Comparisons between FE analysis and experimental failure modes are presented in Figure 17. As shown in Figure 17a, the junctions of the PC-500 specimen have the largest transverse strains and deformations under compression, and will fail first. This is consistent with the observation during the test where the junction regions on the GFRP sheets showed separation failure (see Figure 17b) at ultimate loads. For long column specimens, taking PC-2400 as an example, the global buckling mode is evident, as shown in Figure 17c, and in agreement with the experimental failure mode (see Figure 17d).

**Figure 18:**
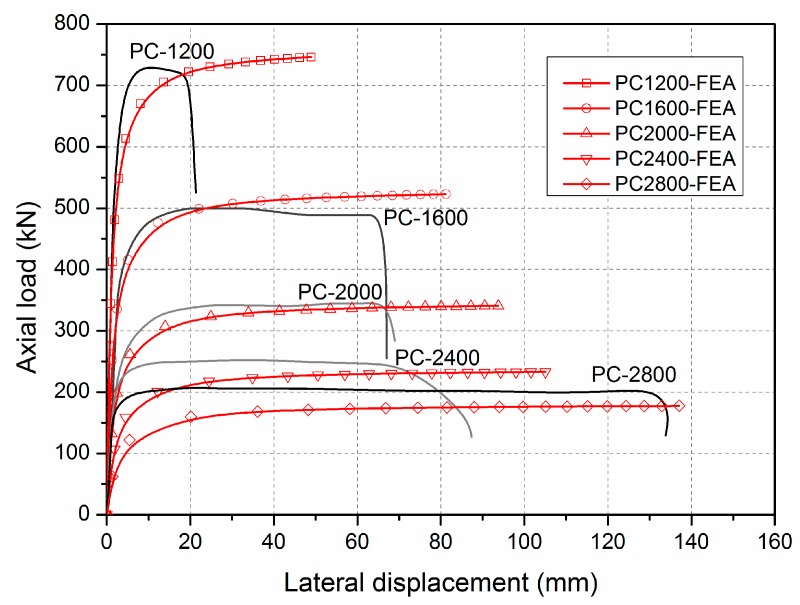
Comparisons between experimental and FE load–lateral displacement curves.

FE results on the load–lateral deformation are presented and compared with experimental results in Figure 18. As shown, the load–lateral displacement curves from FE analysis fit the experimental results well for all specimens in terms of the critical loads and corresponding deformations.

## 5. Design Approach for the Critical Loads

A well-known calculation method of critical loads for pultruded columns under axial compression, proposed by Barbero and Tomblin [21], is as follows:

(7) $$P_{\mathrm{cr}}=(\frac{1+1/{\bar{\lambda }}^{2}}{2c}-\sqrt{\left(\frac{1+1/{\bar{\lambda }}^{2}}{2c}\right){}^{2}-\frac{1}{c{\bar{\lambda }}^{2}}})\cdot P_{L}$$

where *P*_cr_ is the critical load, *P*_L_ is the ultimate axial load of short column or the local buckling load, and *c* is the interaction parameter. Barbero and Tomblin [21] proposed *c* = 0.84 to fit the curve to the experimental mean values. Subsequently, Barbero and De Vivo [22] proposed *c* = 0.65 as a design value. $\bar{\lambda }$ is the non-dimensional slenderness and is defined in Equation (8):

(8) $$\bar{\lambda }=\frac{L_{\mathrm{cr}}}{\pi }\sqrt{\frac{P_{L}}{EI}}$$

where *EI* is the bending stiffness of the column.

A similar calculation method has been developed for GFRP pultruded columns by Puente [27]. This method is shown as follows:

(9) $$P_{\mathrm{cr}}=\frac{1}{\gamma }\cdot \kappa \cdot P_{L}$$

(10) $$\kappa =\frac{1}{\varphi +\sqrt{\varphi^{2}-{\bar{\lambda }}^{2}}}\leq 1$$

(11) $$\varphi =0.5[1+0.12({\bar{\lambda }}^{2}-0.25)+{\bar{\lambda }}^{2}]$$

where the coefficient *γ* is determined by the quality of the material supplied by a manufacturer; it is advisable to use a value of *γ* = 1.2.

**Figure 19:**
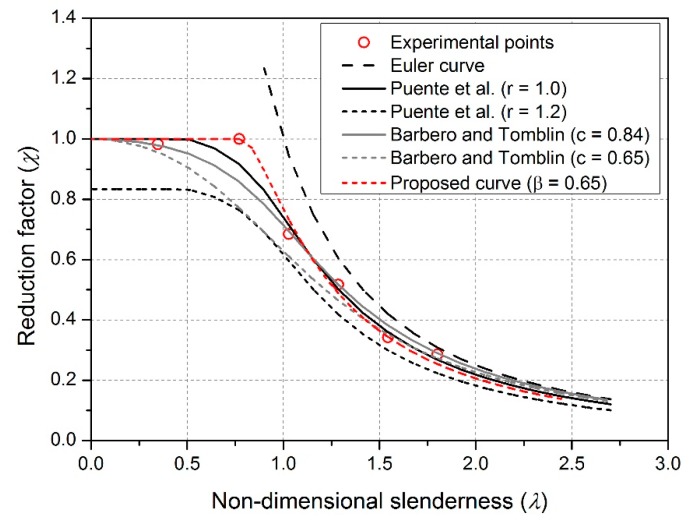
Comparisons between experimental load capacities and design methods.

The predicted critical axial load capacities of the specimens determined by Equations (4)–(9) are shown in Figure 19. The predicted critical axial load capacities are similar to the test values when $\bar{\lambda }$ is greater than 1.0 but are less than the test values when $\bar{\lambda }$ ranges from 0.5 to 1.0. For intermediate length specimens in PC-1200, the relative error (=(predicted value–test value)/test value) between the predicted value and test value ranges from 8.4% to 23.6%. The results indicate that the design methods based on the common pultruded FRP profiles may not be appropriate for the PFWC profile. The reason may lie in the different failure modes for short members: In design methods from [21,22,27], local buckling is the main governing failure mode as the profiles are thin-walled sections; however, for the new PFWC profiles, it shows compressive failure (see specimen PC-500 series) due to the improved sandwich profile with the introduction of wood core.

To propose a new design method for the GFRP/wood composite column, a factor *β* is introduced to improve the Puente’s method, forming Equation (12):

(12) $$\varphi =0.5[1+0.12({\bar{\lambda }}^{2}-\beta )+{\bar{\lambda }}^{2}].$$

Through parametric study, it is found that, when *β* is 0.65, the new proposed load capacities predictions using Equations (9), (10), and (12) are presented in Figure 19 as a proposed curve (red line). As shown, the predicted critical axial load capacities are consistent with the test values, especially for the ones where $\bar{\lambda }$ ranges from 0.5 to 1.0.

## 6. Conclusions

An innovative PFWC profile was fabricated by an improved pultrusion process using E-glass fiber, UPR, and southern pine wood. Axial compression tests were carried out on PFWC column specimens with slenderness ratios ranging from 19.4 to 100.7. Failure modes of specimens with different slenderness ratios were obtained and categorized. Finite element analysis was carried out and compared with experimental results including failure modes and load–displacement curves. The test results for the critical loads of the specimens were compared with the calculated results from existing design equations, and evident deviations were found on intermediate length column specimens. An improved empirical equation was further proposed for estimating the load bearing capacities of such PFWC columns. The following conclusions can be drawn:

1. The improved pultrusion process utilized to fabricate FRP–wood composite columns is automated and continuous, and saves labour in fabricating FRP sandwich structures. The PFWC profiles have superior mechanical properties and have been successfully applied in a whole FRP composite building.
2. The failure modes of the PFWC columns under axial compression include three types: (1) compressive failure on short column specimens, (2) global buckling on slender column specimens, and (3) interaction between compressive failure/global buckling on intermediate column specimens. All failure modes were validated well with load–strain responses of specimens having different slenderness ratios.
3. FE results on the load–lateral displacement curves show satisfactory agreement with the experimental results. The FE results regarding the failure modes especially the short column specimens are close to the experimental results. The critical junction regions of the GFRP sheets experienced severe transverse strain deformations and finally led to junction separation failure.
4. The experimental critical loads of the PFWC specimens at different slenderness ratios were examined using current design equations for pultruded FRP profiles, however evident deviations were found in intermediate length specimens, with non-dimensional slenderness ranging from 0.5 to 1.0. To estimate the load bearing capacities of such PFWC profiles, an improved equation with the introduction of an empirical coefficient was further developed, and good agreement was found between the experimental results and the new proposed equation.

## Figures and Tables

**Table 1:** Material properties of southern pine wood and GFRP.

Materials	Compressive Strength			Compressive Modulus		
	Number of Coupons	Mean Value (MPa)	Mean Squared Error	Number of Coupons	Mean Value (GPa)	Mean Squared Error
southern pine wood	6	51.8	12.79	6	7.4	0.39
GFRP	6	163.2	78.2	6	28.0	5.56

**Table 2:** Dimensions of column specimens.

Specimens	Section *b* × *h* (mm)	*t*/mm	*L*/mm	Slenderness Ratio λ
PC-500	120 × 90	5	500	19.4
PC-1200			1200	43.2
PC-1600			1600	57.6
PC-2000			2000	71.9
PC-2400			2400	86.3
PC-2800			2800	100.7

**Table 3:** Test results of specimens.

Specimens	Slenderness Ratio λ	*P*_u_ (kN)	*P*_cr,t_ (kN)	(*P*_u_*−P*_cr,t_)/ *P*_u_ × 100 (%)	*a* (mm)	Average Value of *P*_cr,t_ (kN)	*P*_cr,E_ (kN)	Relative Error * (%)
PC-1200-1	43.2	730.2	724.2	0.82	0.07	703.9	1042.1	48.05
PC-1200-2		719.8	683.6	5.03	0.25			
PC-1600-1	57.6	500.4	494.8	1.12	0.56	492.7	586.2	18.98
PC-1600-2		523.4	490.6	6.27	1.65			
PC-2000-1	71.9	377.6	366.3	2.99	0.04	362.3	375.2	3.56
PC-2000-2		366.4	358.3	2.21	1.23			
PC-2400-1	86.3	264.1	262.7	0.53	0.74	254.3	260.5	2.44
PC-2400-2		252.2	245.9	2.50	0.15			
PC-2800-1	100.7	209.5	204.1	2.58	0.02	176.9	191.4	8.20
PC-2800-2		173.2	149.7	13.57	2.79			

* Relative error = 100(*P*_cr,E_ – *P*_cr,t_)/*P*_cr,t_ %.

## References

[1] Russo S. (2012). Experimental and finite element analysis of a very large pultruded FRP structure subjected to free vibration. Compos. Struct..

[2] Wu C., Bai Y., Zhao X.L. (2015). Improved bearing capacities of pultruded glass fibre reinforced polymer square hollow sections strengthened by thin-walled steel or CFRP. Thin Wall. Struct..

[3] Zureick A., Scott D. (1997). Short-Term Behavior and Design of Fiber-Reinforced Polymeric Slender Members under Axial Compression. J. Compos. Constr..

[4] Van Den Einde L., Zhao L., Seible F. (2003). Use of FRP composites in civil structural applications. Constr. Build. Mate..

[5] Xie L., Bai Y., Qi Y.J., Wang H. (2019). Pultruded GFRP square hollow columns with bolted sleeve joints under eccentric compression. Compos. Part B Eng..

[6] Kim Y.J., Fam A. (2011). Numerical analysis of pultruded GFRP box girders supporting adhesively-bonded concrete deck in flexure. Eng. Struct..

[7] Nunes F., Correia J.R., Silvestre N. (2016). Structural behavior of hybrid FRP pultruded beams: Experimental, numerical and analytical studies. Thin Wall. Struct..

[8] Ascione L., Berardi V.P., Giordano A., Spadea S. (2015). Pre-buckling imperfection sensitivity of pultruded FRP profiles. Compos. Part B Eng..

[9] Correia M.M., Nunes F., Correia J.R., Silvestre N. (2013). Buckling behavior and failure of hybrid fiber-reinforced polymer pultruded short columns. J. Compos. Constr..

[10] Ascione L., Berardi V.P., Giordano A., Spadea S. (2013). Local buckling behavior of FRP thin-walled beams: A mechanical model. Compos. Struct..

[11] Cricrì G. (2019). Cohesive law identification of adhesive layers subject to shear load—An exact inverse solution. Int. J. Solids. Struct..

[12] İpek G., Arman Y., Çelik A. (2018). The effect of delamination size and location to buckling behavior of composite materials. Compos. Part B Eng..

[13] Perrella M., Berardi V.P., Cricrì G. (2018). A novel methodology for shear cohesive law identification of bonded reinforcements. Compos. Part B Eng..

[14] Xie L., Bai Y., Qi Y.J., Caprani C., Wang H. (2018). Effect of width-thickness ratio on capacity of pultruded square hollow polymer columns. Proc. Inst. Civ. Eng. Struct. Build..

[15] Bai Y., Keller T., Wu C. (2013). Pre-buckling and post-buckling shear failure at web-flange junction of pultruded GFRP beams. Mater. Struct..

[16] Manalo A., Aravinthan T., Fam A., Benmokrane B. (2017). State-of-the-Art Review on FRP Sandwich Systems for Lightweight Civil Infrastructure. J. Compos. Constr..

[17] Fam A., Sharaf T. (2010). Flexural performance of sandwich panels comprising polyurethane core and GFRP skins and ribs of various configurations. Compos. Struct..

[18] Fleck N.A., Sridhar I. (2002). End compression of sandwich columns. Compos. Part A Appl..

[19] Veedu V.P., Carlsson L.A. (2005). Finite-element buckling analysis of sandwich columns containing a face/core debond. Compos. Struct..

[20] Mitra N., Raja B.R. (2012). Improving delamination resistance capacity of sandwich composite columns with initial face/core debond. Compos. Part B Eng..

[21] Barbero E., Tomblin J. (1994). A phenomenological design equation for FRP columns with interaction between local and global buckling. Thin-Walled Struct..

[22] Barbero E.J., DeVivo L. (1999). Beam-column design equations for wide-flange pultruded structural shapes. J. Compos. Constr..

[23] Lane A., Mottram J.T. (2002). Influence of modal coupling on the buckling of concentrically loaded pultruded fiber-reinforced plastic columns. Proc. Inst. Mech. Eng. Part L J. Mater. Design Appl..

[24] Bank L.C. (2006). Composites for construction: Structural design with FRP materials. Pultruded Axial Members.

[25] Hashem Z.A., Yuan R.L. (2000). Experimental and analytical investigations on short GFRP composite compression members. Compos. Part B Eng..

[26] Turvey G.J., Zhang Y. (2006). A computational and experimental analysis of the buckling, postbuckling and initial failure of pultruded GRP columns. Comput. Struct..

[27] Puente I., Insausti A., Azkune M. (2006). Buckling of GFRP columns: An empirical approach to design. J. Compos. Constr..

[28] Bai Y., Keller T. (2009). Shear failure of pultruded fiber-reinforced polymer composites under axial compression. J. Compos. Constr..

[29] McGrath R.D., Murphy J.G., Mitchell P.R., Koppernaes C.C. (1994). Method for Making a Pultruded Panel. U.S. Patent.

[30] Fanucci J., Gorman J., Koppernaes C. (2004). Low Cost Tooling Technique for Producing Pultrusion Dies. U.S. Patent.

[31] Patrick J.F. (2007). Fundamental Characteristics of 3-D GFRP Pultruded Sandwich Panels. Master’s Thesis.

[32] Dawood M., Taylor E., Ballew W., Rizkallad S. (2010). Static and fatigue bending behavior of pultruded GFRP sandwich panels with through-thickness fiber insertions. Compos. Part B Eng..

[33] Zhu D., Shi H.Y., Fang H., Liu W.Q., Qi Y.J., Bai Y. (2018). Fiber reinforced composites sandwich panels with web reinforced wood core for building floor applications. Compos. Part B Eng..

[34] ASTM C365/C365M-16 (2016). Standard Test Method for Flatwise Compressive Properties of Sandwich Cores.

[35] ASTM D695-15 (2015). Standard Test Method for Compressive Properties of Rigid Plastics.

[36] Guades E., Aravinthan T., Islam M.M. (2014). Characterisation of the mechanical properties of pultruded fibre-reinforced polymer tube. Mater. Des..

[37] (2013). ANSYS Mechanical APDL Material Reference Release 15.0. www.ansys.com.

